# Ionising radiation increases permeability of endothelium through ADAM10-mediated cleavage of VE-cadherin

**DOI:** 10.18632/oncotarget.18282

**Published:** 2017-05-30

**Authors:** Sylwia Kabacik, Ken Raj

**Affiliations:** ^1^ Radiation Effects Department, Centre for Radiation, Chemical and Environmental Hazards, Public Health England, Chilton, UK

**Keywords:** permeability, VE-cadherin, ADAM10, cell junctions, ionising radiation

## Abstract

The association between ionising radiation (IR) exposure and risk of cardiovascular diseases (CVD) is well documented, but the underlying mechanism is still poorly understood. As atherosclerotic plaques are the most common cause of CVD, we investigated the effects of IR on one of the critical parameters for atherosclerotic plaque formation – endothelium permeability to macromolecules.

We used endothelial cells from human coronary artery as a model of the endothelial layer. Our results show that exposure of this endothelial layer to IR increased its permeability to macromolecules of various sizes in a dose-dependent manner. Immunofluorescence analysis revealed disruption of cell junctions caused by decreased amounts of two junction proteins, one of which is vascular endothelial cadherin (VE-cadherin). The reduction in the level of this protein was not due to diminished transcription but to protein processing instead. We observed a radiation dose-dependent increase in the cleavage of VE-cadherin by ADAM10. This was not mediated through the canonical VEGF route but was instead accompanied by intra-cellular calcium release. Importantly, inhibition of ADAM10 activity rescued IR-induced permeability.

Our observations demonstrate that exposure to IR activates ADAM10 to cleave VE-cadherin leading to augmented endothelium permeability; a feature that can lead to the development of atherosclerotic plaques and increase the risk of cardiovascular disease.

## INTRODUCTION

The epidemiological association between exposure to ionising radiation (IR) and subsequent development of cardiovascular diseases (CVD) is well-established. There is increased risk of CVD amongst atomic bomb sur*vivo*rs [1], plutonium workers [2] and most notably cancer patients whose hearts or carotid arteries were exposed during radiotherapy [3–6]. In spite of the strength of this association, little is known about the underlying mechanisms of which there may be several, given the range of CVD types.

Atherosclerosis is the main cause of CVD and several studies have indicated that IR exposure accelerates development of atherosclerotic plaques in hypercholesteraemic mice [7–9]. It is well established that IR exposure causes inflammation of endothelium and endothelial hyperplasia [10–12], which both contribute to endothelial dysfunction and consequently have a potential to induce atherosclerosis. However, animal experiments show that anti-inflammatory or anti-coagulant therapies are not successful in preventing IR-induced atherosclerosis [13, 14] suggesting that another mechanism is involved in the process. The two critical steps for the development of atherosclerotic plaques are increased endothelium adhesiveness to monocytes, which encourages monocyte diapedesis, and enhanced permeability of the endothelium to macromolecules [15]. Importantly, IR has been demonstrated to stimulate these two pro-atherosclerotic features of the endothelium. We have previously shown that exposure of cells to IR leads to increased adhesiveness of the endothelium through demethylation of the CD44 promoter; highlighting the potential of non-mutagenic effects of radiation in causing pathology [16]. Others have reported IR to increase permeability of the endothelial monolayer and that of the blood-brain barrier, but the mechanisms that mediate this are not as well elucidated [17, 18]. It is however likely that radiation might affect endothelial permeability by acting on junctions between endothelial cells as they play prominent roles in blood vessel permeability (for a review see [19]). There are two types of cell junctions that regulate the permeability of the endothelium – tight junctions (TJ) and adherens junctions (AJ). Each junction type is formed by different proteins with TJ adhesion being mediated mainly by claudins and occludin, that are connected to the cytoskeleton by tight junction proteins [20]. AJ on the other hand are formed by classical cadherins that are linked to the cytoskeleton by proteins belonging to the catenin family [21]. Endothelial cells originating from different types of vessels exhibit distinctive characteristics in terms of junction organisation and composition of junction proteins [22].

VE-cadherin is an endothelial-specific AJ protein necessary for the proper formation and function of endothelial barriers [23]. Many permeability-inducing agents such as vascular endothelial growth factor (VEGF), interleukin 8, tumor necrosis factor alpha, bradykinin or histamine act by causing phosphorylation of VE-cadherin, leading to its internalisation and often its degradation [24–27]. As is the case with many membrane proteins, VE-cadherin is also very sensitive to cleavage by metalloproteinases and Schulz et al. showed that thrombin induces permeability by a disintegrin and metalloproteinase 10 (ADAM10)-mediated cleavage of VE-cadherin [28].

Here, we report the mechanism by which IR exposure leads to increased permeability of endothelium using human coronary artery endothelial cells (HCAEC) as a model of endothelial layer.

## RESULTS

### IR exposure increases permeability of endothelial layer through disruption of cell junctions

**Figure 1:**
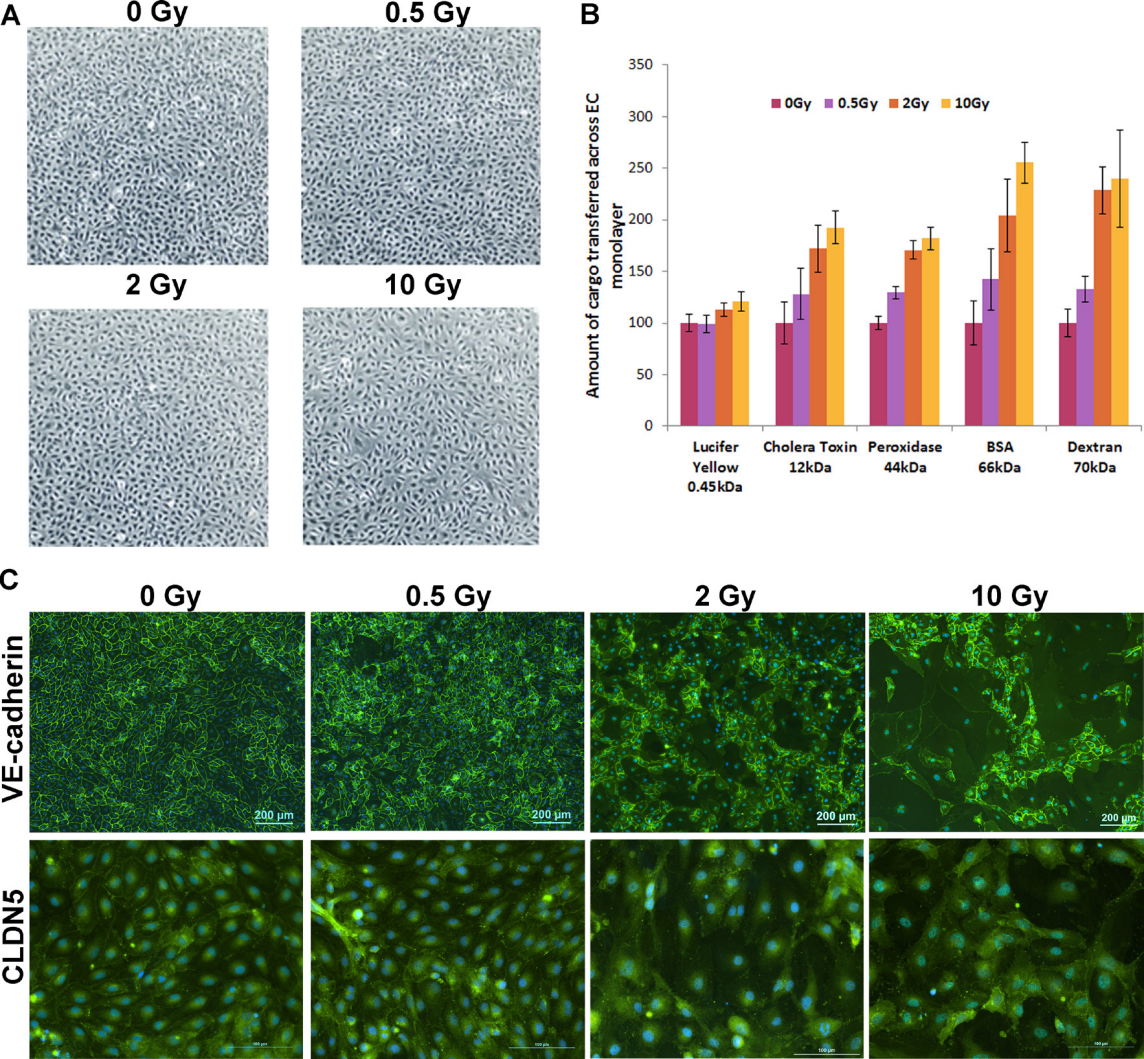
IR exposure increases permeability of endothelial layer through disruption of cell junctions HCAEC were cultured for 7 days following irradiation at doses of 0.5, 2 and 10 Gy of X-ray. (**A**) Phase contrast microscopy pictures of irradiated HCAEC. (**B**) Representative results of the permeability assay. Five macromolecules of different sizes ranging from 0.45 to 70 kDa were used. The amounts transferred across the EC monolayer in an unexposed sample for each of the macromolecules (cargo) were set at 100. Error bars represent standard deviation from three technical replicates. (**C**) Immunofluorescence analysis of cell junctions. VE-cadherin and CLDN5 were stained with fluorescent antibodies (green), DAPI was used for staining nuclei (blue).

To ensure consistency and continued supply of endothelial cells, we generated telomerase-immortalised HCAEC and used them as a model of coronary artery endothelium. HCAEC seeded on transwells were cultured until confluent to allow for a proper cell junction formation, after which they were irradiated with 0.5, 2 or 10 Gy of X-ray. Seven days after irradiation, the monolayer was still intact without visible signs of gaps between cells (Figure 1A). We did not observe significant increase in cell death either 30 h or 7 days post exposure (Supplementary Figure 1A) and only exposure to the highest dose of IR triggered G2 arrest at both time points (Supplementary Figure 1B) suggesting that confluent HCAEC can tolerate substantial doses of IR. Five macromolecules of different sizes ranging from 0.45 kDa to 70 kDa were used to test the permeability of the endothelium. IR exposure significantly increased permeability of all these macromolecules in a dose-dependent manner (Figure 1B).

As cell junctions play a critical physiological role in vessel permeability, we investigated whether IR exposure has an impact on the integrity of cell junctions. Immunofluorescence analysis of VE-cadherin and claudin 5 (CLDN5 – an endothelium specific component of TJ) revealed dose-dependent deterioration of cell junctions which is particularly noticeable for the large cells, which we have previously demonstrated to be senescent (Figure 1C). It is worth noting that we could not detect in HCAEC, another constituent of TJs – occludin (Supplementary Figure 1C), suggesting that it may not be a major component of coronary arterial endothelium TJs.

### IR exposure decreases levels of VE-cadherin and CLDN5 proteins

**Figure 2:**
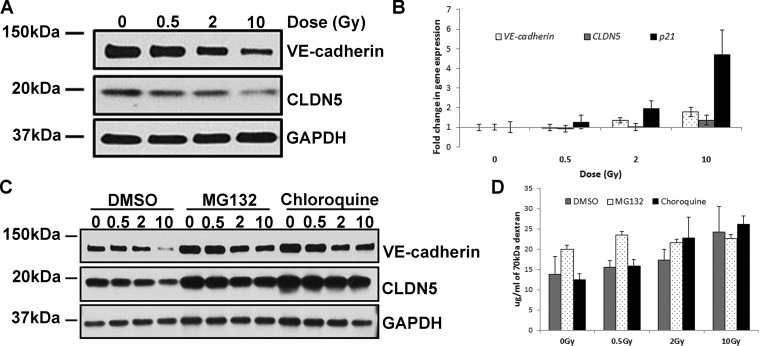
IR exposure decreases levels of VE-cadherin and CLDN5 proteins HCAEC were cultured for 7 days following X-irradiation at doses of 0.5, 2 and 10 Gy. (**A**) Western blot analysis of VE-cadherin and CLDN5; GAPDH was used as a loading control. (**B**) qPCR analysis of *VE-cadherin* and *CLDN5* gene expression. A well-characterised IR-responsive gene - p21 was used as a positive control. Gene expression results were normalised to *HPRT1* endogenous control and represented as fold change in gene expression. Error bars represent standard deviation from three independent experiments. (**C**) Western blot analysis of VE-cadherin and CLDN5 protein level following inhibition of protein degradation. Cells were treated with proteasome inhibitor (MG132), lysosome inhibitor (chloroquine) or vector (DMSO); GAPDH was used as a loading control. (**D**) Representative results of the permeability assay results following inhibition of protein degradation. Cells were treated with proteasome inhibitor (MG132), lysosome inhibitor (chloroquine) or carrier (DMSO) and then subjected to permeability assay with FITC-labelled dextran 70 kDa. Error bars represent standard deviation from three technical replicates.

Western blot analyses revealed a significant decrease in VE-cadherin and CLDN5 protein levels, which are particularly evident after irradiation at higher doses (Figure 2A). In order to test whether the lower levels of these proteins was due to reduced transcription, we measured the levels of *CDH5* (gene encoding the VE-cadherin protein) and *CLDN5* mRNAs. The quantity of transcripts of both genes was slightly increased by IR in a dose-dependent manner, albeit not to the extent of *p21* – a well-known IR-responsive gene (Figure 2B).

Having eliminated transcription repression as a route through which IR reduces VE cadherin and CLDN5 protein levels, we tested whether IR exposure alters the degradation rate of junction proteins. There are two main pathways by which proteins can be degraded: through the proteasome or the lysosome. By using inhibitors that specifically block either of these, we tested to see which, if any of them, mediated IR-induced reduction of junction protein levels. Inhibiting either the proteasome or lysosome increased the basal level of VE-cadherin and CLDN5 proteins, with the latter exhibiting the most apparent augmentation (Figure 2C). Inhibition of lysosome prevented IR-induced decrease of CLDN5 protein level. This however, was not observed when proteasome was inhibited. On the other hand, inhibition of either the lysosome or proteasome did not prevent IR from reducing the level of VE-cadherin.

Since the basal levels of these junction proteins were substantially augmented by the protein degradation inhibitors, we tested whether this would by itself prevent IR from increasing endothelium permeability. To this end we performed permeability experiment using these protein degradation inhibitors. We observed that although inhibiting the proteasome increased the basal level of VE-cadherin and CLDN5 proteins, permeability was not decreased. Instead it increased, and that radiation could further augment this, albeit marginally, in a manner that is not dependent on radiation dose. Inhibition of lysosome, which also increased basal levels of the two junction proteins, neither altered permeability of the non-irradiated endothelium, nor its rise in permeability in response to radiation (Figure 2D). These results suggest that degradation of VE-cadherin or CLDN5 by these two protein degradation pathways is not the mechanism by which IR increases endothelium permeability.

### Inhibition of ADAM10 mitigates IR-induced reduction of VE-cadherin, improves quality of cell junctions and decreases endothelium permeability

**Figure 3:**
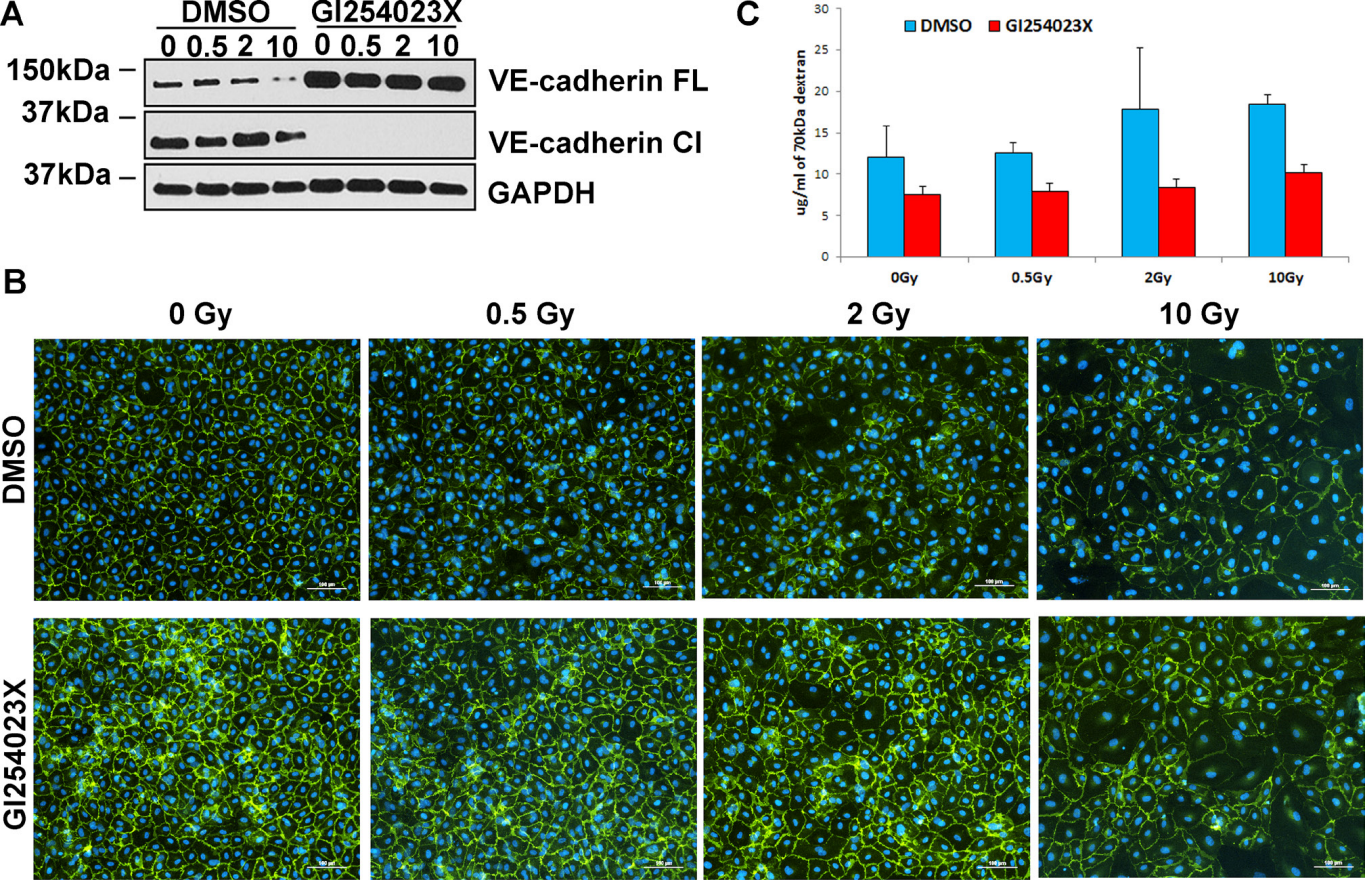
Inhibition of ADAM10 mitigates IR-induced reduction of VE-cadherin, improves quality of cell junctions and decreases endothelium permeability HCAEC were cultured for 7 days following X-irradiation at doses of 0.5, 2 and 10 Gy. (**A**) Western blot analysis of VE-cadherin expression after blocking ADAM10 with GI254023X inhibitor. FL – full length, Cl – cleaved VE-cadherin. GAPDH was used as a loading control. (**B**) Immunofluorescence analysis of cell junctions after treatment with GI254023X inhibitor. VE-cadherin was stained with fluorescent antibodies (green), DAPI was used for staining nuclei (blue). The same exposure times and settings were used to capture control and ADAM10-inhibited samples. (**C**) Representative results of the permeability assay following inhibition of ADAM10. Cells were treated with ADAM10 inhibitor (GI254023X) or carrier (DMSO) and then subjected to permeability assay with FITC-labelled dextran 70 kDa. Error bars represent standard deviation from three technical replicates.

In view of the unlikely involvement of protein degradation as the means by which IR increases endothelial permeability, we turned our attention to protein modification. The 130 kDa VE-cadherin glycoprotein can be cleaved by the enzyme ADAM10, through a process called ectodomain shedding. This releases an approximately 95 kDa soluble ectodomain of VE-cadherin into the extracellular space, while leaving the 35 kDa membrane-bound fragment to be further processed by γ-secretases [28]. Since such VE-cadherin cleavage was reported to increase endothelium permeability [28, 29], we investigated, using an ADAM10-specific inhibitor GI254023X, whether ADAM10-mediated cleavage of VE-cadherin is responsible for IR-induced permeability. As would be expected, even in the absence of radiation, GI254023X blocked VE-cadherin cleavage completely, resulting in a substantial increase in the level of full-length VE-cadherin. Interestingly, this inhibitor also prevented reduction in VE-cadherin protein level after irradiation (Figure 3A), which is reflected by retention of VE cadherin at the cell junctions even after exposure to the highest radiation doses (Figure 3B). Importantly, inhibition of ADAM10 prevented the rise in permeability that would otherwise have occurred when these cells were irradiated (Figure 3C), indicating that ADAM10-mediated cleavage of VE-cadherin is responsible for increased permeability following IR exposure.

### IR exposure stimulates ADAM10 activity

**Figure 4:**
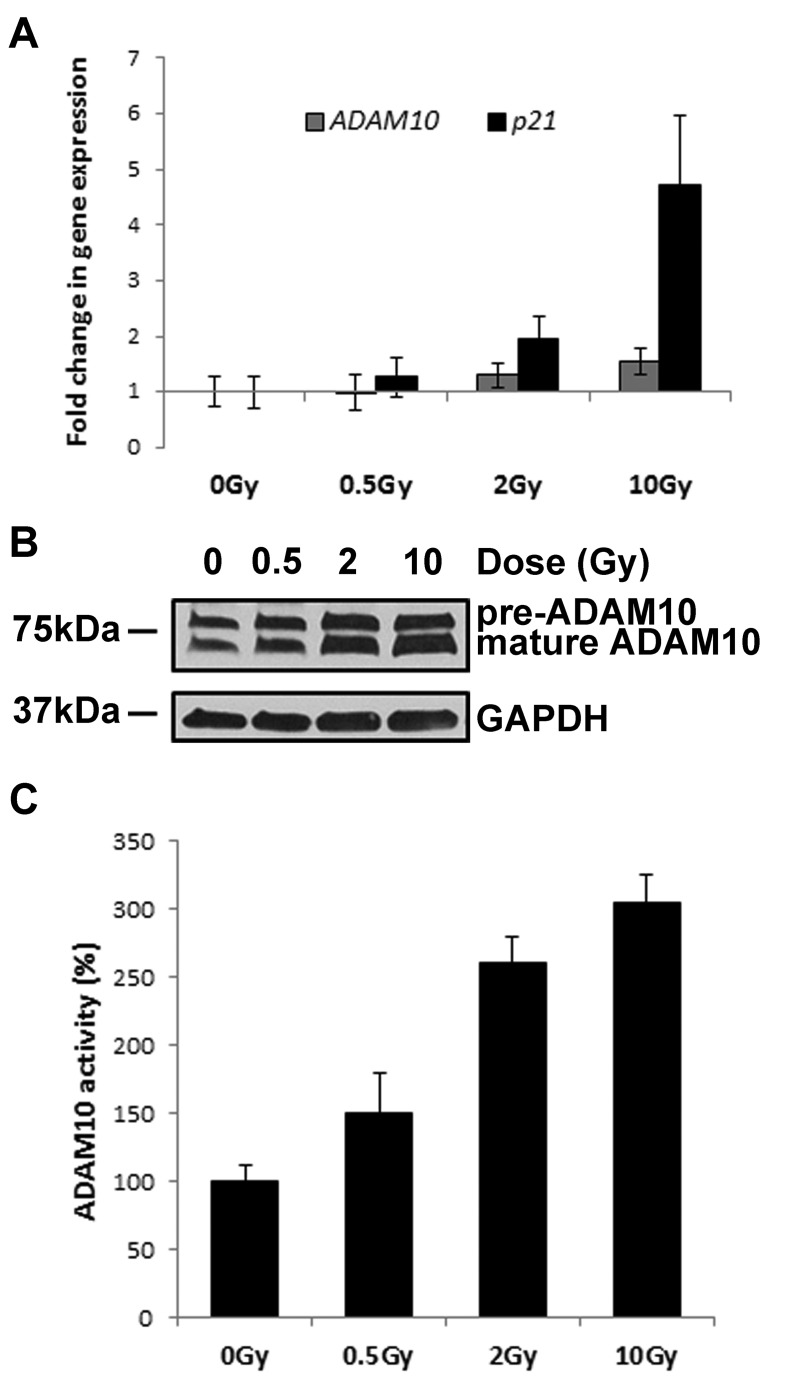
IR exposure stimulates ADAM10 activity HCAEC were cultured for 7 days following irradiation with 0.5, 2 and 10 Gy of X-ray. (**A**) qPCR analysis of *ADAM10* gene expression. A well-characterised IR-responsive gene - p21 was used as a positive control. Gene expression results were normalised to *HPRT1* endogenous control and represented as fold change in gene expression. Error bars represent standard deviation from three independent experiments. (**B**) Western blot analysis of ADAM10 protein; GAPDH was used as a loading control. (**C**) Measurement of ADAM10 activity following IR exposure. An ADAM10-specific substrate which emits fluorescence upon cleavage was added to live cells. Following 1 h incubation at 37°C fluorescence was measured in the medium. Error bars represent standard deviation from three technical replicates.

To test whether IR-exposure does indeed increase ADAM10 activity, as suggested by the experiments described above, we first tested the effect of IR on ADAM10 expression using qPCR analysis. Although we observed a radiation dose-dependent increase in ADAM10 transcript level, the magnitude of increase was very modest (Figure 4A). ADAM10 protein is synthetized as an inactive precursor of about 84 kDa which is subsequently cleaved by a furin endopeptidase or proprotein convertase 7, to produce a mature and active 68 kDa enzyme [30]. We observed that IR exposure elevated the levels of both precursor and mature ADAM10 protein (Figure 4B); however, the mature form was increased to a greater extent than the precursor, suggesting that IR induces ADAM10 maturation. Finally, as raised protein level does not necessary mean that its function is increased, we measured ADAM10 activity in live cells using ADAM10 specific substrate which fluoresces upon cleavage. We found that IR exposure significantly stimulated ADAM10 activity in a dose-dependent manner, reaching 3-fold augmentation after exposure to the highest dose, proving that IR exposure does indeed increase ADAM10 activity in HCAEC (Figure 4C).

### IR-induced permeability is independent of VEGF signalling

Radiation-induced augmentation of ADAM10 activity has not been previously reported, and hence the underlying mechanism is unknown. There are however, publications which demonstrate that VEGF, by binding to its receptor (vascular endothelial growth factor receptor 2 – VEGFR2), induces the expression and the activity of ADAM10, resulting in cleavage of VE-cadherin via extracellular signal-regulated kinase 1 (ERK1) signalling [31]. Furthermore, it is reported that VEGF can also induce permeability by phosphorylating the SRC protein, which can in turn directly or indirectly phosphorylate VE-cadherin causing accelerated endocytosis of the later [24]. Moreover, *VEGF* gene expression was reportedly induced by IR exposure [32] offering a plausible mechanism for IR-induced permeability.

**Figure 5:**
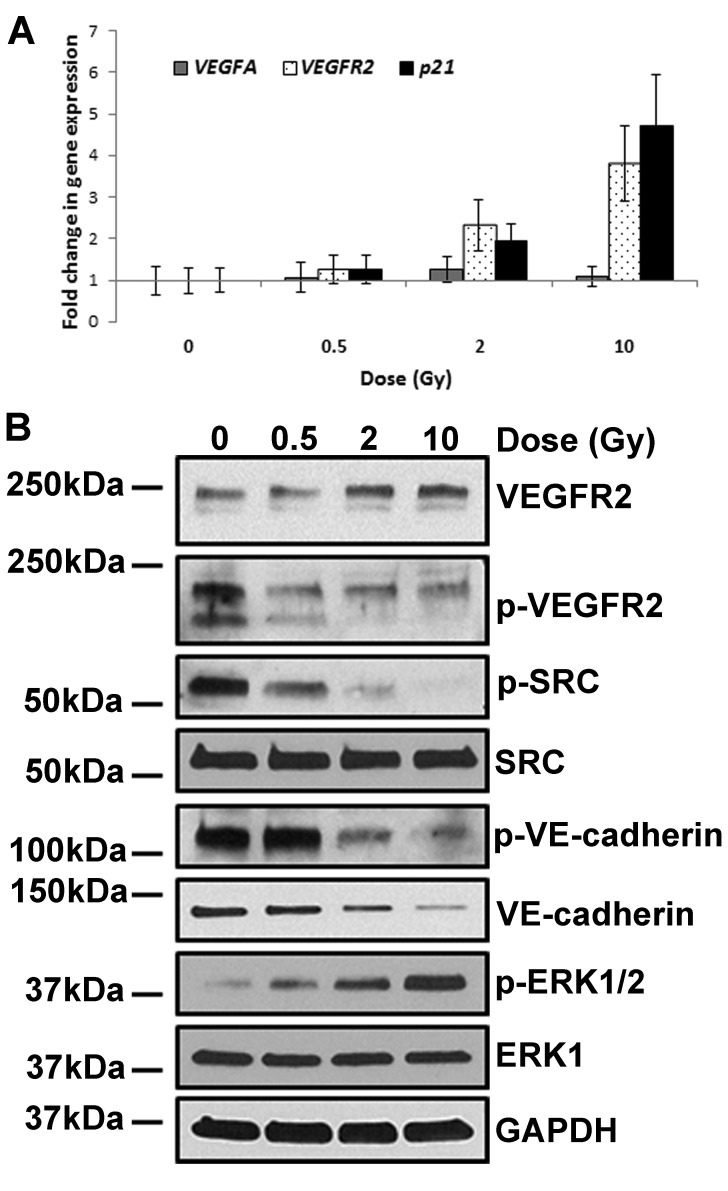
IR-induced permeability is independent of VEGF signalling HCAEC were cultured for 7 days following irradiation with 0.5, 2 and 10 Gy of X-ray. (**A**) qPCR analysis of *VEGFA* and *VEGFR2* gene expression. A well-characterised IR-responsive gene - p21 was used as a positive control. Gene expression results were normalised to *HPRT1* endogenous control and represented as fold change in gene expression. Error bars represent standard deviation from three independent experiments. (**B**) Western blot analysis of proteins involved in VEGFA signalling; GAPDH was used as a loading control.

Our analyses however, did not detect any significant alteration of *VEGF* transcript level in irradiated cells (Figure 5A). Although the quantity of mRNA and protein of VEGFR2 were increased in these cells (Figure 5B), the phosphorylated active form did not rise concomitantly, but was instead markedly decreased. This observation is all the more surprising as the level of phosphorylated ERK1/2 kinase, which was reportedly stimulated by the VEGF signalling pathway, was augmented in these irradiated cells (Figure 5B). The unexpected inactivation of VEGF signalling by radiation is further confirmed by the decrease in the level of phosphorylated SRC protein. Collectively, these observations support the conclusion that (i) IR represses VEGF signalling in HCAEC, (ii) activation of VEGF signalling is not the means by which radiation exerts its effects on endothelium permeability and (iii) the radiation-induced rise in phosphorylated ERK1/2 that we observed must occur through another means that is independent of VEGF activation.

### IR exposure increases intracellular calcium concentration

**Figure 6:**
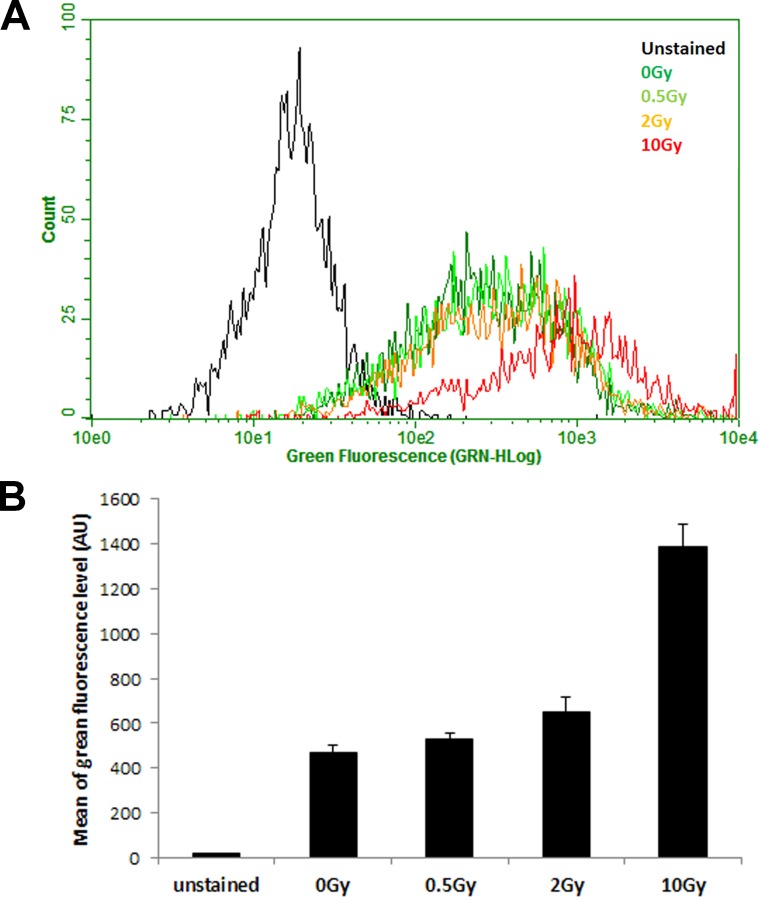
IR exposure increases intracellular calcium concentration HCAEC were cultured for 7 days following irradiation with 0.5, 2 and 10 Gy of X-ray, and then loaded with intracellular calcium indicator Fluo-4, AM. (**A**) Histogram of green fluorescence representing intracellular calcium level analysed by flow cytometry. (**B**) Arithmetic mean of green fluorescence in each sample. Error bars represent standard deviation from three technical replicates measured in duplicates.

After excluding VEGF signalling as a potential mechanism by which IR induces permeability of endothelium, we turned our attention to calcium signalling, as it is well established that calcium influx stimulates ADAM10 activity [33–35]. By using cell permeable dye, Fluo-4 AM which emits green fluorescence upon binding Ca2+ ions, we measured calcium levels in HCAEC 7 days post IR exposure by flow cytometry. We observed a radiation dose-dependent increase in intracellular calcium concentration (Figure 6) which mirrors that of ADAM10 activity, lending support to the notion that radiation-induced activation of ADAM10 is mediated by increases in intracellular calcium levels.

## DISCUSSION

CVD is the leading cause of deaths worldwide and while modern radiotherapy techniques aim to reduce dose to the heart and major arteries during the treatment, it is not always possible especially for left breast [36] or head and neck cancer [5]. The epidemiological data from breast cancer patients shows that the risk of CVD started to increase 5 years after radiotherapy [3], which presents a window of opportunity for potential countermeasures. It is obvious that understanding the mechanism by which IR increases risk of CVD is essential in order to develop effective treatment, especially as there is evidence suggesting that IR-induced atherosclerosis might be very different from its age-related counterpart [13, 14].

Here we investigated the mechanism by which IR increases permeability of endothelium, which is one of the critical steps for atherosclerotic plaque formation. We discovered that IR exposure had detrimental and long-lasting effect on endothelial cell junctions (Figure 1B and 1C). IR substantially reduced the level of VE-cadherin and CLDN5 proteins – important constituents of AJ and TJ respectively (Figure 2A). IR-induced permeability could not be prevented by blocking protein degradation through either the proteasome or the lysosome (Figure 2D), even though this substantially increased the levels of both proteins and prevented IR-induced drop in CLDN5 (Figure 2C). This may suggest that VE-cadherin plays a dominant role in IR-induced permeability of arterial endothelium. This notion is consistent with reports demonstrating that hepatocytes lacking E-cadherin – another AJ protein – form TJ at a slower rate, suggesting that AJs promote TJ organisation [37]. VE-cadherin can also control TJ by increasing transcription of the *CLDN5* gene [38]. It is possible that with reduced VE-cadherin level, TJ cannot function properly even if CLDN5 level is elevated by other means.

VE-cadherin plays a central role in vascular permeability (reviewed in [39]). Phosphorylation of VE-cadherin and its subsequent internalisation is a mode of action for many permeability-inducing factors [24–27]. Additionally, VE-cadherin shedding by ADAM10 was implicated in inflammation-induced breakdown of endothelial barrier functions in human dermal microvascular endothelial cells [29]. Conversely, stabilisation of VE-cadherin at cell junction blocks leukocyte extravasation and decreases vascular permeability [40].

Here, we have shown that IR induces endothelium permeability through stimulation of ADAM10 activity which leads to increased cleavage of VE-cadherin (Figures 3 and 4). Although it has been shown that ADAM10-dependent cleavage of VE-cadherin can be stimulated by VEGF signalling in an ERK1-dependent manner [31], our data does not support VEGF signalling as a mode of action for IR-induced permeability pathway (Figure 5). Even though, phosphorylated ERK1/2 kinase was elevated by IR exposure it is unlikely that VEGF signalling is responsible for its activation (Figure 5B).

**Figure 7:**
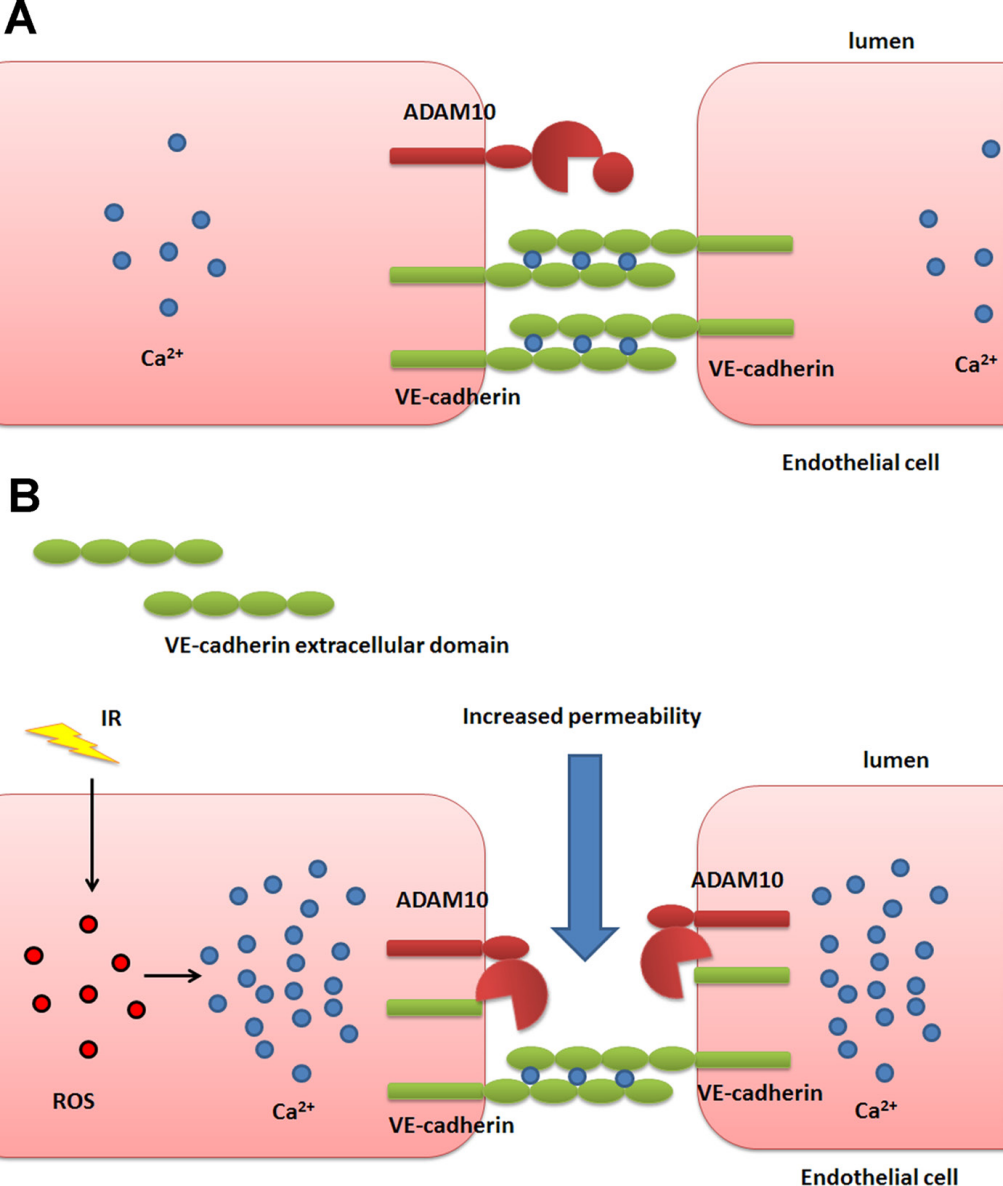
Schematic overview of the proposed mechanism of IR-induced endothelial permeability (**A**) In healthy cells VE-cadherin proteins from adjacent endothelial cells form adherens junctions to regulate endothelial permeability. (**B**) Irradiation of cells triggers production of ROS, which causes a rise in the concentration of intracellular calcium. This in turn stimulates the activation of ADAM10 which cleaves VE-cadherin, weakening cell junctions and increasing endothelial permeability.

We observed considerable increase in ADAM10 protein level following IR exposure despite the fact that ADAM10 transcription was only marginally increased. While it is intuitive to expect the amounts of transcripts to change in parallel magnitude with that of proteins, this expectation is often not met, because most protein quantities are actually regulated at the level of translation, as was only recently appreciated [41]. Our results also show that after IR exposure, the increase in the matured form of ADAM10 is greater than that of the precursor (Figure 4B) demonstrating enhanced processing of the latter. It is well-established that calcium influx stimulates ADAM10 maturation and activity probably through disruption of the association between ADAM10 and calmodulin [33–35]. Indeed, IR exposure increased intracellular calcium concentration in HCAEC in a dose-dependent manner (Figure 6) offering a plausible explanation for IR-induced stimulation of ADAM10 activity. This proposition is further strengthened by the fact that calcium influx has been reported to stimulate activation of the ERK1/2 pathway [37, 38] which we also observe to rise considerably following IR exposure (Figure 5B). Interestingly, reactive oxygen species (ROS) are reportedly able to stimulate endothelium hyperpermeability by increasing cytosolic calcium concentration [42]. Since IR is known to increase ROS production in cells [43], we propose the following model: IR increases ROS in cells, triggering a rise in calcium levels, which activates ADAM10 to cleave VE-cadherin, resulting in the increase of endothelium permeability (Figure 7).

It is worth noting the more important and broader implication of these observations. It is clear that IR-induced activation of ADAM10, which is reproducible and readily detectable within 7 days post-irradiation, is mediated by a mechanism that is highly inconsistent with random mutations. Instead, it is reminiscent of the non-mutational route we previously described for radiation-induced adhesiveness of endothelial cells [16]. Collectively, radiation, through non-mutational means, can induce adhesiveness at the apical surface of endothelial cells and at the same time disrupt lateral junctions between endothelial cells. This synergistic mode of action, which compromise endothelial functions, would be expected to increase the risk of atherosclerotic plaque formation and consequently risk of CVD development. It is important to appreciate the principle that radiation can induce potentially pathological changes independently of its mutagenic effect. This is especially relevant in the context of health protection, which would benefit from further characterisation of such effects of radiation on other tissues and pathologies.

Furthermore, IR-induced stimulation of ADAM10 activity may have consequences beyond increased permeability of endothelium. ADAMs are promiscuous enzymes that usually process many different proteins involved in various aspects of vascular biology (reviewed in [44]). Among ADAM10 substrates are: interleukin 6 receptor [45], tumor necrosis factor alpha [46] – both having pro-inflammatory properties, VEGFR2 [31], betacellulin [47] – both having a role in controlling permeability, CXC-chemokine ligand 16 [48], fractalkine [49] – both facilitating leukocyte transmigration. It would appear that increasing ADAM10 activity is an effective and efficient way by which IR establishes an inflammatory and permissible environment for atherosclerotic plaque formation. Indeed, animal experiments show that IR-induced atherosclerotic plaques are more inflammatory, richer in metalloproteinases and more prone to rupture than their age-induced counterparts [7].

The increased ADAM10 activity triggered by IR exposure may also have detrimental effects on established atherosclerotic plaques, as mature plaques already have higher ADAM10 expression than early plaques or healthy endothelium [31]. Moreover, it has been shown that unsaturated free fatty acids and enzymatically-modified low density lipoprotein which accumulate near atherosclerotic lesions increase ADAM10-mediated substrate cleavage [50]. Any additional effects IR exert on ADAM10 could act synergistically with these factors to exacerbate the loss of permeability control of the endothelial layer over existing plaques, rendering the plaques more inflammatory and prone to rupture.

Finally, pinpointing ADAM10 as a cause of IR-induced endothelium permeability offers a new possibility for prevention of IR-induced CVD, especially when animal experiments show that anti-inflammatory or anti-coagulant therapies are not successful in preventing IR-induced atherosclerosis [13, 14].

To summarise, we have identified ADAM10-mediated shedding of VE-cadherin as an underlying cause of IR-induced endothelium permeability. We have shown that IR exposure stimulates ADAM10 activity probably by increasing intracellular calcium concentration in a dose-dependent manner and blocking ADAM10 with specific inhibitor is sufficient to restore endothelium barrier following exposure.

## MATERIALS AND METHODS

### Cell culture

Primary Human Coronary Artery Endothelial Cells (HCAEC) from a 19 years old male were purchased from European Collection of Authenticated Cell Cultures (cat. 300–05a). HCAEC were immortalised with retrovirus construct containing hTERT gene. We have previously tested that telomerase immortalisation does not influence cellular responses to IR [16, 51]. Following selection, cells were cultured in Cornig dishes (Appleton Woods) coated with fibronectin (Sigma-Aldrich, cat. F0895) in MesoEndo Cell Growth Medium (Sigma-Aldrich, cat. 212–500) in a humidified incubator at 37°C and 5% CO_2_. Cells were passed when confluent with the medium changed every other day. For all experiments cells of fewer than 10 population doublings were used.

### Irradiations

HCAEC were irradiated when confluent with doses of 0, 0.5, 2 and 10 Gy of X-ray at room temperature using an A.G.O. HS X-ray system (Aldermaston, Reading, UK) (output 13 mA, 250 kV peak, 0.5 Gy/min) and cultured for 7 days until further analysis. In order to inhibit various cellular pathways, 0.1% DMSO (Sigma-Aldrich, cat. D2438), 50 μM Chloroquine (Sigma-Aldrich, cat. C6628) or 10 μM GI254023X (Sigma-Aldrich, cat. SML0789) were added to cells 16 h before processing. 50 μM MG132 (Sigma-Aldrich, cat. M7449) was added 2 h before processing. No gamma secretase inhibitor was used as HCAEC with low population doubling have weak gamma secretase activity (our own unpublished data).

### Permeability assay

50,000 cells were seeded on a matrigel-coated transwells (Appleton Woods, cat. CC403) and irradiated when confluent. Cells were cultured for 7 days with media changed every other day. Just before permeability assay, medium was completely removed from top and bottom compartments and 0.5 ml of fresh medium was added to the bottom well and 0.5 ml of medium with fluorescent cargo at concentration 1 mg/ml was loaded into the top compartment. Transwells were incubated at 37°C for 2 h then the medium from bottom compartment was collected and the fluorescence level was measured in triplicates by using Synergy HT Multi-Mode Microplate Reader (BioTek UK). Peroxidase assay was carried out according to the manufacturer’s recommended protocol. Briefly, one tablet of SIGMA FASR Buffer with Urea and peroxidase and another of SIGMA FAST OPD were dissolved in 20 ml of water. 25 ul of the prepared substrate was mixed with 25 ul of culture media from the outer transwell and the colour development was measured using a plate reader at 495nm. The cargos used in the assays are listed in Table 1.

**Table 1:** The substrates used in permeability experiments

Fluorescent cargo	Molecular mass	Supplier	Catalogue number
**Lucifer Yellow**	0.46 kDa	Sigma-Aldrich	L0259
**Cholera toxin subunit B (recombinant), Alexa Fluor^®^ 488 conjugate**	At neutral pH, the 11.4 kDa B subunit exists as a 57 kDa pentamer	Thermo Fisher Scientific	C-34775
**FITC-dextran**	70 kDa	Sigma-Aldrich	46945
**Albumin from Bovine Serum (BSA), Alexa Fluor^®^ 488 conjugate**	66 kDa	Thermo Fisher Scientific	A13100
**Horseradish peroxidase**	44 kDa	Sigma-Aldrich	P6782

### Immunofluorescence assay

HCAEC were seeded on fibronectin-coated coverslips and irradiated when confluent with the stated doses. Seven days post-irradiation the adherent cells were rinsed twice with Hanks’ Balanced Salt Solution (HBSS) with added calcium and magnesium to preserve cell junctions (Thermo Fisher Scientific, cat. 14025050) and fixed in formalin for 10 min. Then cells were permeabilised with 0.1% Trition X-100 in HBSS for 10 minutes followed by, blocking in 2% Foetal Calf Serum in HBSS three times, 10 min each. Coverslips were then incubated with primary antibodies for VE-Cadherin and CLDN5 for 1 h at room temperature. Next, coverslips were subjected to three 10 min washes with 2% Foetal Calf Serum in HBSS and incubated with Alexa conjugated secondary antibodies for 1 h at room temperature. Coverslips were then washed again three times, 10 mins each in FCS/HBSS and mounted on slides with Vectashield with DAPI (Vector Laboratories, cat. H-1500). Imaging was performed by using Nikon Eclipse Ti inverted microscope, data analysis was performed with NIS- Elements AR Microscope Imaging Software (Nikon UK). The primary antibodies used for immunofluorescence experiments are listed in Table 2.

**Table 2:** The antibodies used in immunofluorescence experiments

Antibody	Dilution	Supplier	Catalogue number
**VE-Cadherin**	1:100	Santa Cruz Biotechnology	sc-9989
**CLDN5**	1:1000	Abcam	ab131259

### Protein extraction

Seven days after irradiation, cells were rinsed twice with ice-cold HBSS with added calcium and magnesium to preserve cell junctions and collected by scraping. Cells for protein analysis were centrifuged and pellets were frozen at –80°C.

Frozen cell pellets were lysed in 1% SDS, 50 mM Tris, pH 8.0 buffer with added Halt(TM) Protease and Phosphatase Inhibitor Cocktail (100x) (Thermo Fisher Scientific, cat. 10137963) and proteins were quantified by Pierce™ BCA Protein Assay Kit (Thermo Fisher Scientific, cat. 23227) according to manufacturer’s protocol.

### Western blot

Protein lysates were separated on 4–20% Mini-PROTEAN TGX Gels (Bio-Rad, cat. 456–1096) and transferred onto PVDF membrane (Bio-Rad, cat. 170–4156) using semidry Trans-Blot^®^ Turbo™ Transfer System (Bio-Rad) and high molecular weight standard protocol. Membranes were blocked in 5% TBS-T milk at room temperature for a minimum of 1 hour with gentle rocking. The primary antibodies used are listed in Table 3. Blots were incubated with primary antibodies at room temperature overnight, with gentle rocking. Blots were washed three times with TBS-T and incubated with secondary antibodies for 1 h at room temperature. The secondary antibodies used are also listed in Table 3. After washing three times in TBS-T, blots were imaged using Immobilon Western Chemiluminescent HRP Substrate (Millipore, cat. WBKLS0500)

**Table 3:** The antibodies used in western blot experiments

Antibody	Dilution	Protein lysate	Supplier	Catalogue number
VE-cadherin	1:1000	10 μg	Santa Cruz Biotechnology	sc-9989
VE-cadherin [pY658]	1:1000	20 μg	Invitrogen	44-1144G
CLDN5	1:30000	1 μg	Abcam	ab131259
VEGFR2	1:20000	5 μg	Cell Signaling Technology	9698
VEGFR2 (Tyr996)	1:1000	20 μg	Cell Signaling Technology	2474
SRC	1:10000	15 μg	Abcam	ab109381
SRC pY419	1:5000	20 μg	Abcam	ab185617
ERK1	1:1000	10 μg	Bethyl	A302-060A
PhosphoERK1/2 (T202/Y204, T185/Y187)	1:1000	10 μg	Bethyl	A303-608A
ADAM10	1:10000	10 μg	ProSci	2051
OCLN	1:1000	50μg	Abcam	ab31721
GAPDH	1:2000	1 μg	Santa Cruz Biotechnology	sc-25778
GAPDH	1:10000	10 μg	Santa Cruz Biotechnology	sc-25778
GAPDH	1:30000	15 – 50 μg	Santa Cruz Biotechnology	sc-25778
donkey anti-mouse IgG-HRP	1:5000		Santa Cruz Biotechnology	sc-2314
donkey anti-rabbit IgG-HRP	1:10000		Santa Cruz Biotechnology	sc-2313

### RNA extraction

Seven days after irradiation, cells were rinsed twice with ice-cold HBSS and collected by scraping. Cells were resuspended in 1ml of RNA Later (Sigma-Aldrich) and stored at –80°C until further processing. Total RNA was prepared by using Direct-zol™ RNA MiniPrep (Zymo Research, cat. R2050) according to manufacturer’s protocol; DNA contamination was removed by DNase I provided with the kit. RNA quantity was assessed by Nanodrop ND2000 and RNA quality was assessed by using TapeStation 2200 instrument (Agilent Technologies). All samples had RINe values above 8.0.

### Gene expression

Reverse transcriptase reactions were performed using High Capacity cDNA Reverse transcription kit, (Thermo Fisher Scientific, cat. 4368814) according to manufacturer’s protocol using 300 ng of total RNA per 50 µl reaction. For each sample a “-RT” control reaction which did not contain the enzyme, was prepared to assess level of genomic DNA contamination. Real-time quantitative PCR was performed using RotorGene Q. All reactions were run in triplicate using PerfeCTa SYBR^®^ Green SuperMix (Quanta Biosciences, cat. 95054–100), primers at 300 nM concentration each and 0.3 µl of cDNA in 10 µl reaction volume. The primers used are listed in Table 4. Cycling parameters were 2 min at 95°C, then 45 cycles of 10 s at 95°C and 60 s at 60°C. Data was collected and analysed by RotorGene Q analysis software using ddCt method and hypoxanthine phosphoribosyltransferase 1 (HPRT1) as a reference gene.

**Table 4:** Primers sequences used in qPCR experiments

Primer	Sequence
HPRT1-F	TCAGGCAGTATAATCCAAAGATGGT
HPRT1-R	AGTCTGGCTTATATCCAACACTTCG
VE-cad-F	ATGTAGGCAAGATCAAGTCAAG
VE-cad-R	CCTCTCAATGGCGAACAC
CLDN5-F	GACCTTCTCCTGCCACTA
CLDN5-R	CGCTCTGCCTATGGAAAC
VEGFA-F	ACCATGCCAAGTGGTCCCA
VEGFA-R	GCTGCGCTGATAGACATCCAT
VEGFR2-F	TAGAAGACTCAGGCATTGT
VEGFR2-R	CTTTTGCACAGCCAAGAA
ADAM10-F	TGCTGAATGGATTGTGGCTCAT
ADAM10-R	AAAGTGCCTGGAAGTGGTTTAG
p21-F	GCAGACCAGCATGACAG
p21-R	TAGGGCTTCCTCTTGGA

### ADAM10 activity

SensoLyte^®^ 520 ADAM10 Activity Assay Kit (Cambridge Bioscience, cat. ANA72226) was used to measure ADAM10 activity in live cells. 50,000 cells were seeded on fibronectin-coated coverslips and irradiated when confluent with appropriate doses. Cells were cultured for 7 days with media changed every other day. After 7 days, the media was removed from coverslips and 200 μl of fresh media containing 100 x diluted ADAM10 substrate was added to cells. A calibration curve was prepared using recombinant ADAM10 protein diluted in fresh medium. Both cells and calibration curve were incubated for 1 h at 37°C and then the fluorescence was measured in triplicates by using Synergy HT Multi-Mode Microplate Reader (BioTek UK).

### Apoptosis assay and cell cycle profiling

HCAEC were cultured on 6 cm^2^ plates until confluent then irradiated with appropriate doses of IR. Cells were cultured for 7 days with media changed every other day. After 7 days, cells were rinsed twice with HBSS and tripsinised. At this point cells from each radiation dose were divided in half for apoptosis assay and cell cycle profiling. Apoptosis was measured by using FITC Annexin V Apoptosis Detection Kit I (BD Biosciences, cat. 556547) according to manufacturer’s protocol. Unstained control, FITC only and PI only controls were prepared for setting up the gates. The samples were analysed on Guava easyCyte HT flow cytometer (Merk Millipore) using apoptosis program.

Cells for cell cycle profiling were permeablised and fixed for 1 h in cold 70% ethanol. Following this, cells were centrifuged and re-suspended in 500 µl of HBSS containing 100 ng/µl of RNase A (Sigma-Aldrich) and 40 ng/µl of propidium iodide (Sigma-Aldrich) and incubated at 37°C for 30 min. The samples were analysed on Guava easyCyte HT flow cytometer (Merk Millipore) using cell cycle program.

### Measurement of intracellular calcium level

HCAEC were seeded on fibronectin-coated 4-well plates and irradiated when confluent with the stated doses. Seven days post-irradiation the cells were rinsed twice with HBSS with added calcium and magnesium to preserve cell junctions (Thermo Fisher Scientific, cat. 14025050) and loaded with 5 μM Fluo-4 AM (Thermo Fisher Scientific, cat. F14201) in HBSS with added calcium and magnesium at 37°C for 1 h. After incubation HCAEC were washed twice with HBSS without calcium and magnesium and trypsinised. The samples were analysed on Guava easyCyte HT flow cytometer (Merk Millipore). For each dose 3 samples were used and measured in duplicates

## SUPPLEMENTARY MATERIALS FIGURE



## Abbreviations

ADAM10: A disintegrin and metalloproteinase domain-containing protein 10
AJ: adherens junctions
CLDN5: claudin 5
CVD: cardiovascular diseases
GAPDH: glyceraldehyde-3-phosphate dehydrogenase
HBSS: Hanks’ balanced salt solution
HCAEC: Human Coronary Artery Endothelial Cells
HPRT1: hypoxanthine phosphoribosyltransferase 1
IR: ionising radiation
OCLN: occluding
ROS: reactive oxygen species
TJ: tight junctions
VE-cadherin: vascular endothelial cadherin
VEGF: vascular endothelial growth factor
VEGFR2: vascular endothelial growth factor receptor 2
