# Supplementary material for: Ionising radiation increases permeability of endothelium through ADAM10-mediated cleavage of VE-cadherin
Source: Oncotarget. 2017 May 30;8(47):82049–63. doi: 10.18632/oncotarget.18282 (PMC5669869; doi:10.18632/oncotarget.18282)
Supplement: Supplementary file 1 [file oncotarget-08-82049-s001.pdf]

Ionising radiation increases permeability of endothelium through ADAM10-mediated cleavage of VE-cadherin

Supplementary Materials

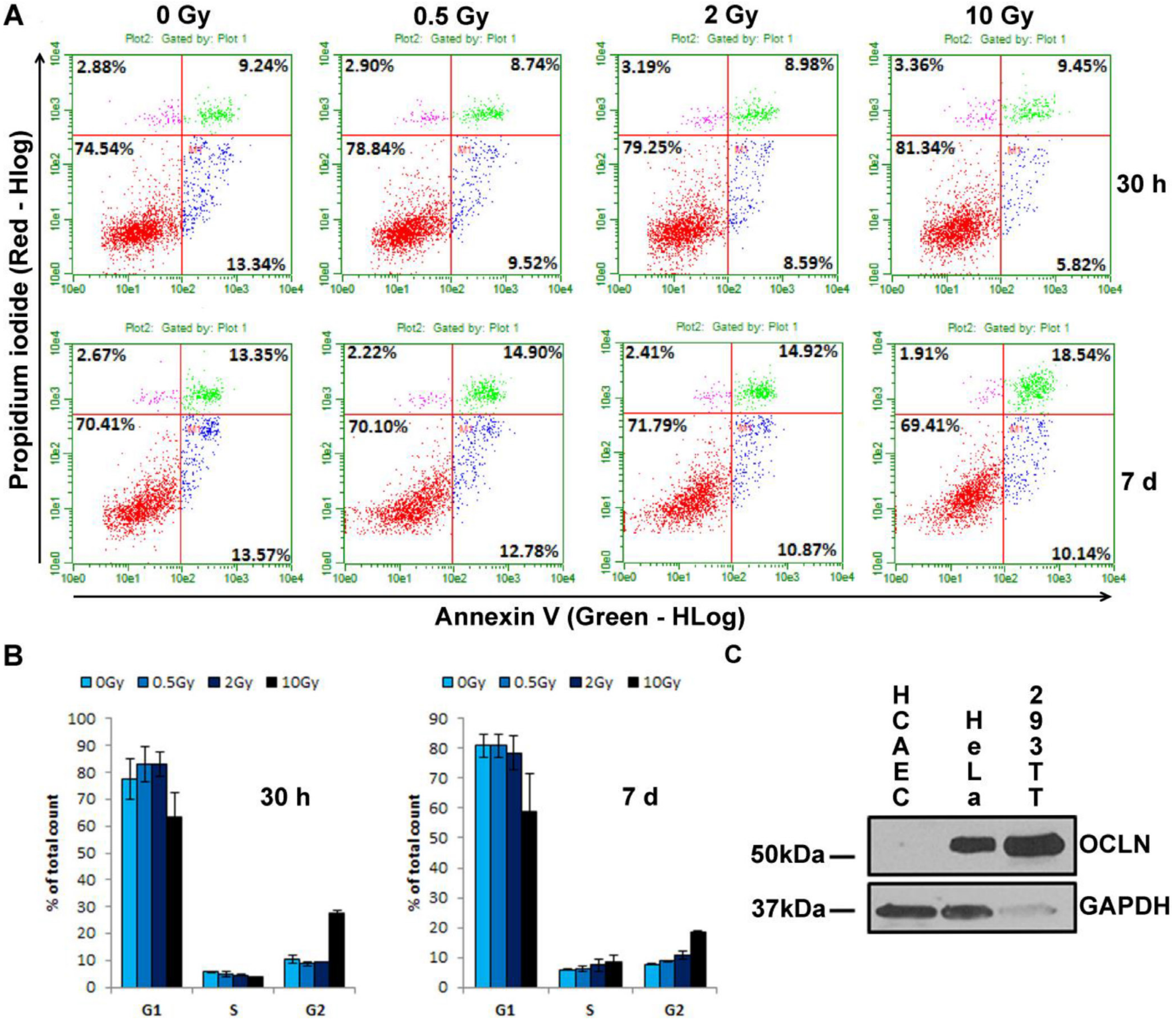

Suplemmentary Figure 1: (**A**) Induction of apoptosis following IR exposure. Following irradiation HCAEC were collected 30 h or 7 d after exposure and stained with FITC-labelled antibody against annexin V and propidium iodide. Labelled cells were analysed by flow cytometry. Unstained control, FITC-only and propidium iodide -only labelled samples were used to set up the gates. LL quadrant represents population of healthy cells, LR–apoptotic cells, UR–late apoptosis, UL–dead cells. Numbers represent percentage of total gated cells in each quadrant. (**B**) Cell cycle profiling following IR exposure. Following irradiation HCAEC were collected 30 h or 7 d after exposure, permeablised and labelled with propidium iodide. Labelled cells were analysed by flow cytometry. Error bars represent standard deviation from two independent experiments. (**C**) Western blot analysis of OCLN protein. 50 µg of protein lysates from HCAEC, HeLa and 293TT cells were separated on a polyacrylamide gel. GAPDH was used as a loading control. Of note, the GAPDH expression is much lower in 293TT cells than in HCAEC or HeLa cells.
